# Cytotoxicity of the Essential Oil of Fennel (*Foeniculum vulgare*) from Tajikistan

**DOI:** 10.3390/foods6090073

**Published:** 2017-08-28

**Authors:** Farukh Sharopov, Abdujabbor Valiev, Prabodh Satyal, Isomiddin Gulmurodov, Salomudin Yusufi, William N. Setzer, Michael Wink

**Affiliations:** 1 Department of Pharmaceutical Technology, Avicenna Tajik State Medical University, Rudaki 139, Dushanbe 734003, Tajikistan; shfarukh@mail.ru (F.S.); valizoda83@gmail.com (A.V.); gulmurodov@mail.ru (I.G.); salomudin@mail.ru (S.Y.); 2 Institute of Pharmacy and Molecular Biotechnology, Heidelberg University, Im Neuenheimer Feld 364, Heidelberg 69120, Germany; 3 Department of Chemistry, University of Alabama in Huntsville, Huntsville, AL 35899, USA; prabodhsatyal@gmail.com (P.S.); setzerw@uah.edu (W.N.S.)

**Keywords:** *Foeniculum vulgare*, essential oil, *trans*-anethole, anise aldehyde, cytotoxicity, cluster analysis

## Abstract

The essential oil of fennel (*Foeniculum vulgare*) is rich in lipophilic secondary metabolites, which can easily cross cell membranes by free diffusion. Several constituents of the oil carry reactive carbonyl groups in their ring structures. Carbonyl groups can react with amino groups of amino acid residues in proteins or in nucleotides of DNA to form Schiff’s bases. Fennel essential oil is rich in anise aldehyde, which should interfere with molecular targets in cells. The aim of the present study was to investigate the chemical composition of the essential oil of fennel growing in Tajikistan. Gas chromatographic-mass spectrometric analysis revealed that the main components of *F. vulgare* oil were *trans*-anethole (36.8%); α-ethyl-*p*-methoxy-benzyl alcohol (9.1%); *p*-anisaldehyde (7.7%); carvone (4.9%); 1-phenyl-penta-2,4-diyne (4.8%) and fenchyl butanoate (4.2%). The oil exhibited moderate antioxidant activities. The potential cytotoxic activity was studied against HeLa (human cervical cancer), Caco-2 (human colorectal adenocarcinoma), MCF-7 (human breast adenocarcinoma), CCRF-CEM (human T lymphoblast leukaemia) and CEM/ADR5000 (adriamycin resistant leukaemia) cancer cell lines; IC_50_ values were between 30–210 mg L^−1^ and thus exhibited low cytotoxicity as compared to cytotoxic reference compounds.

## 1. Introduction

Fennel (Arpabodiyon, local Tajik name), *Foeniculum vulgare* Miller, an important member of the Apiaceae, is widely used for flavouring foods and beverages due to its pleasant spicy aroma [1,2]. In traditional medicine, the plant and its essential oil have been extensively used as carminative, digestive, galactogogue and diuretic and to treat respiratory and gastrointestinal disorders [1]. It is also used as a constituent in cosmetic and pharmaceutical products [3]. The essential oil of *F. vulgare*, in particular anethole, exhibits antispasmodic, carminative, anti-inflammatory, estrogenic and anti-microbial activities [4]. In vitro, fennel oil possesses antioxidant [5,6], antimicrobial [7], insecticidal [8], antithrombotic [9] and hepatoprotective activities [2]. Furthermore, the essential oil of fennel exhibits in vitro anticancer activity [10,11,12]. The in vitro cytotoxic, genotoxic, and apoptotic activities of estragole were suspected to induce hepatic tumors in susceptible strains of mice [10].

Anethole is toxic in high concentrations [4]. Because of their lipophilic properties, the secondary metabolites of essential oils are able to penetrate cytoplasmic membranes by free diffusion. This process can affect membrane fluidity and permeability, transport, ion equilibrium and membrane potential [13], leading to cell death by apoptosis and necrosis [11].

The essential oil of fennel is rich in secondary metabolites, which carry reactive substituents (among them carbonyl groups) in their ring structures or side chains. Aldehydes are generally long-lived and electrophilic compounds, they can react with molecular targets which carry free amino groups, such as of amino acid residues in proteins or of nucleotides in DNA to form Schiff’s bases [14]. Aldehyde-containing essential oils often exhibit cytotoxicity [15,16] by reacting with cellular nucleophiles, including proteins and nucleic acids [13,17].

The chemical composition of the essential oil of *F. vulgare* from different geographical locations has been extensively studied [6,18,19,20]. According to these studies, the major components of fennel oil are *trans*-anethole, estragole, fenchone, and limonene depending on the chemotype [21,22,23]. The aim of the present study was to investigate the chemical composition of the essential oil of fennel growing in Tajikistan (Central Asia) and to explore cytotoxic activity against different human cancer cell lines. The biological activity and chemical composition of *F. vulgare* oil from Tajikistan have not been previously reported.

## 2. Materials and Methods

### 2.1. Plant Material

The aerial parts of *F. vulgare* plants were collected from the Varzob region, Tajikistan on 29 July 2016. A voucher specimen of the plant material was deposited at the Department of Pharmaceutical Technology, Avicenna Tajik State Medical University under accession number TD2016-24. The material was completely dried and hydrodistilled using a Clevenger-type apparatus for 3 h to give an essential oil yield of 0.5%.

### 2.2. Gas-Liquid Chromatography-Mass Spectrometry (GLC-MS)

The essential oil from *F. vulgare* oil was analyzed by GLC-MS using an instrument (GCMS-QP2010 Ultra, Shimadzu, Tokyo, Japan) operated in the EI mode (electron energy = 70 eV), scan range = 3.0 scans s^−1^. The GC column was ZB-5 fused silica capillary with a (5% phenyl)-polymethyl siloxane stationary phase a film thickness of 0.25 mm. The carrier gas was helium with a column head pressure 551 kPa and flow rate of 1.37 mL min^−1^. Injector temperature was 250 °C and the ion source temperature was 200 °C, increased in temperature rate 2 °C min^−1^ to 260 °C. The GC oven temperature program was programmed from 50 °C initial temperature, increased at a rate of 2 °C min^−1^ to 260 °C. A 5% *w*/*v* solution of the sample in CH_2_Cl_2_ was prepared and 0.1 µL was injected in splitting mode (30:1).

Identification of the oil components was based on their retention indices determined by reference to a homologous series of *n*-alkanes (Kovats RI), and by comparison of their mass spectral fragmentation patterns with those reported in the literature [24] and stored on the MS library (NIST 11 (National Institute of Standards and Technology, Gaithersburg, MD, USA), WILEY 10 (John Wiley & Sons, Inc., Hoboken, NJ, USA), FFNSC version 1.2 (Shimadzu Corp., Tokyo, Japan)). The percentages of each component are reported as raw percentages based on total ion current without standardization (set 100%).

### 2.3. Antioxidant Activity

The antioxidant activity of the essential oils was evaluated by 2,2-diphenyl-1-picrylhydrazyl (DPPH), 2,2′-azinobis-(3-ethylbenzthiazoline-6-sulfonic acid) (ABTS) and ferric reducing antioxidant power (FRAP) assays. DPPH, ABTS and FRAP assays were performed as described earlier by us [25,26].

### 2.4. Cytotoxicity

The potential cytotoxicity of the fennel essential oil against of the five human tumor cell lines (HeLa, Caco-2, MCF-7, CCRF-CEM and CEM/ADR5000) were determined by the MTT assay. The cells were seeded at a density of 2 × 10^4^ cells/well (HeLa, Caco-2, MCF-7) and 3 × 10^4^ cells/well (CCRF-CEM and CEM/ADR5000). The essential oil was serially diluted in media in the presence of DMSO at concentrations between 10 mg/L and 5 g/L; 100 μL of each concentration was applied to the wells of a 96-well plates. Cells were incubated with the essential oil for 24 h (HeLa, Caco-2, MCF-7) and 48 h (CCRF-CEM and CEM/ADR5000) before the medium was removed and replaced with fresh medium containing 0.5 mg/mL 3-(4,5-dimethylthiazol-2-yl)-2,5-diphenyltetrazolium bromide (MTT). The formazan crystals produced were dissolved in DMSO 4 h later; the absorbance was measured at 570 nm with a Biochrom Asys UVM 340 Microplate Reader (Cambridge, UK).

### 2.5. Hemolytic Activity

The hemolytic activity was investigated by incubation of serially diluted fennel essential oil in phosphate-buffered saline with red blood cells (human O+). The hemolytic activity assay was performed as described earlier [27].

### 2.6. Hierarchical Cluster Analysis

A total of 68 chemical compositions of *F. vulgare* essential oils, including the sample from this study in addition to 66 compositions obtained from the published literature [5,6,7,9,23,28,29,30,31,32,33,34,35,36,37,38,39,40,41,42,43,44,45] were used to carry out the cluster analysis using the XLSTAT software, version 2015.4.01. The essential oil compositions were treated as operational taxonomic units (OTUs) and the percentages of 34 of the most abundant essential oil components (*trans*-anethole, limonene, estragole, fenchone, α-pinene, α-phellandrene, *p*-anisaldehyde, β-phellandrene, β-pinene, *exo*-fenchyl acetate, *p*-cymene, myrcene, (*E*)-β-ocimene, camphor, 10-nonacosanone, piperitenone oxide, sabinene, neophytadiene, *cis*-anethole, *trans*-dihydrocarvone, γ-terpinene, carvone, phytol, 1,8-cineole, *iso*-isopulegol, *trans*-β-terpineol, *endo*-fenchyl acetate, camphene, carvacrol, apiole, *o*-cymene, δ-3-carene, linalool, and thymol) were used to establish the chemical relationships of the *F. vulgare* essential oil samples using the agglomerative hierarchical cluster (AHC) method. Pearson correlation was selected as a measure of similarity, and the unweighted pair-group method with arithmetic average (UPGMA) was used for definition of the clusters.

### 2.7. Microscopic Observation

The images of the treated or untreated CCRF cells were obtained and photographed using a by fluorescence microscopy (BZ-9000, Keyence, Osaka, Japan) in order to investigate morphological changes.

### 2.8. Data Analysis

The experiments were repeated three times. IC_50_ values were calculated using a four parameter logistic curve (Sigma Plot 11.0 (SYSTAT Software, San Jose, CA, USA)). The data are represented as means ± standard deviations. The results of statistical test were determined by using Sigma Plot 11.0 software and also by using the statistical function *t*-test in Microsoft Excel. A *p* value below 0.05 was considered to represent statistical significance.

## 3. Results and Discussion

### 3.1. Chemical Composition

The essential oil of *F. vulgare* was analyzed by gas-liquid chromatography—mass spectrometry (GLC-MS). Thirty components were identified representing 97.7% of total oil composition (Table 1). Oxygenated terpenoids were the dominant compounds of the essential oil of *F. vulgare.*

**Scheme 1:**
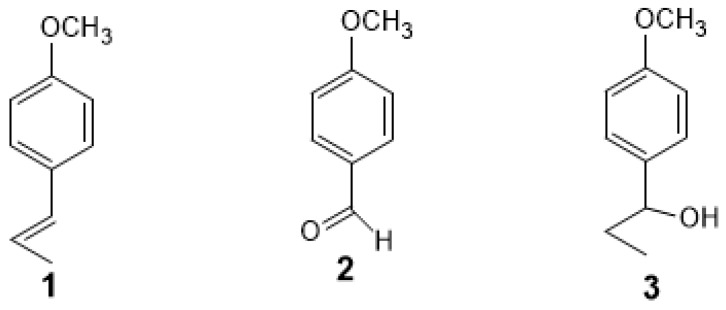
Structures of main components of the essential oil *Foeniculum vulgare.*

The major components were *trans*-anethole (**1**) (36.8%), *p*-anisaldehyde (**2**) (7.7%), α-ethyl-*p*-methoxybenzyl alcohol (**3**) (9.1%), carvone (4.9%), 1-phenylpenta-2,4-diyne (4.7%) and fenchyl butanoate (4.2%). The main three compounds (*trans*-anethole, *p*-anisaldehyde, α-ethyl-*p*-methoxybenzyl alcohol) are both ethers, having methoxy functional groups (Scheme 1).

In accordance with previously published data, **1** is the main component [1,46], its content varying from 5.0 to 85%. However, estragole [47,48], fenchyl acetate [7] and limonene [6] have also been reported as main components of the fennel oil from other origins. Fennel essential oil is known as a source for anethole [49].

*F. vulgare* is subdivided into three main chemotypes according to their relative compositions: (1) estragole chemotype; (2) estragole/anethole chemotype and (3) anethole chemotype [34]. The essential oil of *F. vulgare* from Tajikistan thus belongs to the anethole chemotype, which is widely distributed [47].

### 3.2. Cluster Analysis

**Figure 1:**
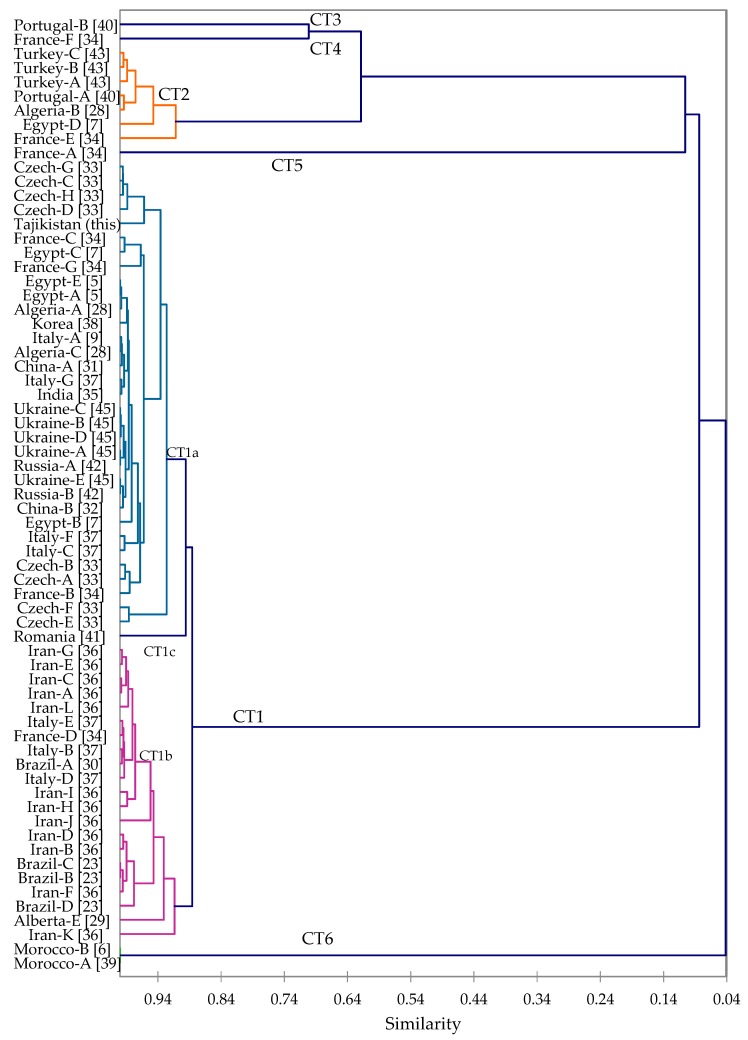
Dendrogram obtained from the agglomerative hierarchical cluster analysis of 68 *Foeniculum vulgare* essential oil compositions. (CT1) anethole-rich chemotype, (CT1a) anethole chemotype, (CT1b) anethole/limonene chemotype, (CT1c) anethole/camphor chemotype, (CT2) estragole chemotype, (CT3) estragole/α-phellandrene chemotype, (CT4) anethole/estragole/α-pinene chemotype, (CT5) α-phellandrene chemotype, and (CT6) limonene/β-pinene/myrcene chemotype.

In order to place the chemical composition of Tajik *F. vulgare* into context with previous investigations, a hierarchical cluster analysis was carried out using the essential oil composition from this study in conjunction with compositions from 66 samples previously reported in the literature [5,6,7,9,23,28,29,30,31,32,33,34,35,36,37,38,39,40,41,42,43,44,45]. The cluster analysis (Figure 1) reveals the major chemotype of *F. vulgare* to be an anethole-rich chemotype (CT1), which includes the sample from Tajikistan. There is also an estragole-rich chemotype (CT2), represented by seven samples, and several chemotypes represented by only one or two samples each: an estragole/α-phellandrene chemotype (CT3), an anethole/estragole/α-pinene chemotype (CT4), an α-phellandrene chemotype (CT5), and a limonene/β-pinene/myrcene chemotype (CT6). The anethole-rich cluster can be subdivided into three chemotypes: an anethole chemotype (CT1a, including the sample from Tajikistan), an anethole/limonene chemotype (CT1b), and an anethole/camphor chemotype (CT1c) represented by a single sample from Romania (see Figure 1).

### 3.3. Antioxidant Activity

The investigation of antioxidant activity of essential oils as lipophilic secondary metabolites became an interesting aspect of food and pharmaceutical research. Synthetic food additives are increasingly replaced with plant-based natural ingredients, due to their safety, effectiveness and consumer acceptance [50]. In general, fennel as an edible and medicinal plant represents an interest through the neutralization of reactive oxygen species in order to prevent the damage of protein, lipid, and DNA which are supposed to be the main reason for cell aging, oxidative stress-originated diseases (cardiovascular and neurodegenerative diseases), and cancer.

The essential oil of fennel exhibits low antioxidant activity as compared to the positive control, caffeic acid. The results of the DPPH, ABTS and FRAP analyses are represented Table 2.

The concentration of 50% inhibition (IC_50_) was the parameter used to compare the DPPH and ABTS radical scavenging activity. A lower IC_50_ (for DPPH and ABTS) and higher FRAP values indicate higher antioxidant activity. IC_50_ values for the antioxidant activity were 15.6 mg mL^−1^ (DPPH) and 10.9 mg mL^−1^ (ABTS). The IC_50_ values of the known antioxidant substance—caffeic acid—were 0.0017 mg mL^−1^ for DPPH and 0.0011 mg mL^−1^ for ABTS, respectively. Ferric reducing antioxidant power (FRAP) were equivalent to 193.5 µM Fe(II)/mg for oil and 2380 µM Fe(II)/mg for caffeic acid. In agreement with our results, it was reported that the an IC_50_ value of DPPH radical scavenging activity of *Foeniculum vulgare* essential oil was 15.3 mg mL^−1^ [7]. According to the authors [7], fennel essential oil reacts with free radicals as a primary antioxidant and, therefore, it may limit free-radical damage occurring in the human body. In our previous paper, we reported the antioxidant activity of pure essential oil components, including the main component of the essential oil of fennel (*trans*-anethole). It shows weak antioxidant activity. We assume that the phenolic substances (carvacrol (2.1%) and thymol (1.0%)) are responsible for the observed antioxidant activity. These data are in agreement with previously reported data [25]. However, it is known that the bioactivity of plant extracts is due to the entire composition of the extract [51,52].

### 3.4. Cytotoxicity

The cytotoxicity of the oil was tested against HeLa, Caco-2, MCF-7, CCRF-CEM and CEM/ADR5000 cancer cell lines (Table 3). IC_50_ values were 207 mg L^−1^ for HeLa, 75 mg L^−1^ for Caco-2, 59 mg L^−1^ for MCF-7, 32 mg L^−1^ for CCRF-CEM, and 165 mg L^−1^ for CEM/ADR5000 cell lines. As compared to the positive control doxorubicin, the essential oil exhibits low cytotoxicity. Doxorubicin, an anthracycline antitumor antibiotic is a hydrophilic drug, and shows broad spectrum anticancer activity [53]. The cytotoxicity of *F. vulgare* oil is most likely due to the lipophilic properties of essential oil and alkylating properties of the major components *trans*-anethole and *p*-anisaldehyde. Caco-2 and CEM/ADR5000 overexpress the ABC transporter p-gp which can actively pump out any lipophilic compound that has entered the cell by free diffusion [54]). Thus, both cell lines are rather insensitive towards lipophilic cytotoxic agents. In contrast, the parent cell line CCRF-CEM should be sensitive. We also suspect that some components of the essential oil are may be substrates for p-gp, as IC_50_ values were higher in CEM/ADR5000 cells.

**Figure 2:**
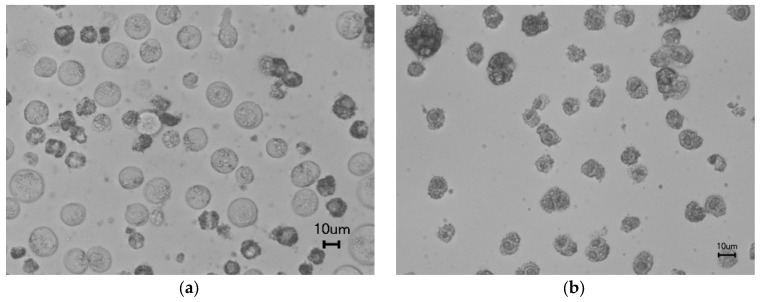
The images of untreated (**a**) and treated (**b**) CCRF cells with essential oil of *Foeniculum vulgare.*

To better understand the mechanism of action of *F. vulgare* essential oil, we have investigated its hemolytic effect. The result of hemolytic activity indicates that the oil is able to lyse the cell membrane albeit with a rather high IC_50_ value of 1100 mg L^−1^ (Table 3). Moreover, in order to investigate the effect of essential oil on the cell morphology, the images of untreated and treated CCRF cells with essential oil were captured by fluorescence microscope. The images are illustrated in Figure 2.

Obtained images indicate that the essential oil can change the morphology of cells. Results of both hemolysis and microscopic investigation indicate that essential oil also affects the integrity of cell membranes. This is in agreement with many of the reported data [55].

In addition, *trans*-anethole, the main component of the essential oil, was examined for its cytotoxicity in RC-37 cells. Its IC_50_ value was 100 mg L^−1^ [56]. The incubation of hepatocytes with anethole caused a cell death accompanied by losses of cellular ATP and adenine nucleotide pools [57]. Anethole shows apoptotic activity, as it can damage DNA [58]. Thus anethole could be responsible for the overall cytotoxicity of the essential oil in our study.

## 4. Conclusions

The essential oil of fennel contains several bioreactive secondary metabolites, such as aldehydes. The oil apparently affects the stability of biomembranes and interacts with molecular targets, such as proteins and DNA, which causes a low cytotoxicity.

## Figures and Tables

**Table 1:** Chemical composition of the essential oil of *Foeniculum vulgare* according to a GLC-MS analysis.

Compounds	% *	RT **	RI ***
*trans*-Anethole	36.8	36.022	1286
α-Ethyl-*p*-methoxybenzyl alcohol	9.10	54.059	1569
*p*-Anisaldehyde	7.73	33.825	1254
Carvone	4.87	33.119	1243
1-Phenylpenta-2,4-diyne	4.75	35.875	1283
Fenchyl butanoate	4.23	46.653	1448
Neomenthol	3.62	28.027	1170
(2*E*)-Dodecenal	3.44	47.807	1467
β-Ethyl-*p*-methoxybenzyl alcohol	3.27	54.498	1577
*trans*-Thujone	2.95	24.404	1118
Fenchone	2.75	22.408	1089
Carvacrol	2.15	36.802	1297
Linalyl acetate	1.88	33.503	1249
Unidentified	1.39	42.318	1380
(*E*)-Chrysanthenyl acetate	1.38	34.015	1256
Thymol	1.03	36.255	1289
Fenchyl isobutanoate	1.03	47.726	1465
(*E*)-β-Terpineol	1.00	28.642	1179
Linalool	0.75	23.139	1100
*cis*-Thujone	0.74	23.624	1107
(*E*)-Dihydrocarvone	0.64	30.422	1204
Unidentified	0.64	48.021	1470
Geranial	0.56	34.747	1267
Myrtenyl acetate	0.54	36.160	1288
*exo*-Fenchyl acetate	0.48	32.269	1231
Penta-1,3-diynylbenzene	0.46	40.505	1353
Dill ether	0.44	29.148	1186
Methylchavicol (=estragole)	0.42	29.973	1198
Unidentified	0.30	53.885	1567
Caryophyllene oxide	0.25	54.759	1581
Camphor	0.23	26.445	1147
*iso*-Menthone	0.10	27.050	1156
1-Hexadecene	0.08	48.270	1474
**Terpene hydrocarbons:**	**5.21**		
**Oxygenated terpenoids:**	**85.78**		
**Others:**	**6.70**		
**Total identified:**	**97.67**		

* Total peak area was set to 100%; ** Retention time; *** Kovats retention index in ZB-5 column.

**Table 2:** Antioxidant activity of the essential oil *Foeniculum vulgare* as determined by the ABTS, DPPH, and FRAP assays *.

Sample	DPPH IC_50_ (g L^−1^)	ABTS IC_50_ (g L^−1^)	FRAP µM Fe(II)/mg of Samples
*Foeniculum vulgare*	15.6 ± 1.1 **	10.9 ± 0.4 **	194 ± 18 **
*trans*-Anethole	23.4 ± 0.1 **	35.6 ± 0.1 **	104 ± 5.2 **
Caffeic acid	0.0017 ± 0.0002 ***	0.0011 ± 0.0002 ***	2380 ± 46 ***

* The data are represented as means ± standard deviations; ** significant at *p* < 0.0025; *** significant at *p* < 0.0001.

**Table 3:** Cytotoxicity of the essential oil of *Foeniculum vulgare*.

Sample	HeLa	Caco-2	MCF-7	CCRF-CEM	CEM/ADR 5000	RBC
	IC_50_, mg L^−1^ *					
*Foeniculum vulgare*	207 ± 13 **	75 ± 4 **	59 ± 5 **	32 ± 1 **	165 ± 15 ***	1100 ± 50 **
*Doxorubicin*	4.5 ± 0.6 **	1.1 ± 0.1 **	1.3 ± 0.3 **	0.25 ± 0.2 **	1.4 ± 0.4 **	-

* The data are represented as means ± standard deviations; ** significant at *p* < 0.0006; *** significant at *p* < 0.001.
